# Comparison of generic and specific instruments to assess the quality of life in patients with melasma

**DOI:** 10.1186/s12874-022-01599-5

**Published:** 2022-04-20

**Authors:** Silmara Meneguin, Ioana Bitencourt Mourão, Camila Fernandes Pollo, Helio Amante Miot, Miriane Garuzi, Cesar de Oliveira

**Affiliations:** 1 Nursing Department, Botucatu Medical School, São Paulo State University (Unesp), Botucatu, Brazil; 2 Department of Dermatology, Botucatu Medical School, São Paulo State University (Unesp), Botucatu, Brazil; 3 Department of Epidemiology & Public Health, University College London, London, UK

**Keywords:** Melasma, Quality of life, Psychometry, Questionnaire

## Abstract

**Objective:**

To compare the psychometric performance of a generic and specific instruments in assessing melasma-related quality of life.

**Methods:**

A cross-sectional study was conducted with 150 patients with melasma attending an outpatient dermatology clinic of a public hospital in São Paulo state, Brazil. Data were collected using a questionnaire containing sociodemographic and clinical data as well as the generic WHOQOL-BREF, and the dermatological-specific Skindex-16 and HRQ-Melasma.

**Results:**

The overall internal consistency of the domains of the three instruments was ≥ 0.7. A strong positive correlation was identified between the Skindex-16 and HRQ-Melasma domains (0.68-0.78). Item-response theory showed that most Skindex-16 and HRQ-Melasma domains were more informative than WHOQOL-BREF.

**Conclusion:**

The three instruments for assessing QOL tested presented good psychometric performance, with satisfactory internal consistency values. Only the two dermatological instruments, however, demonstrated a strong correlation between the domains that assess social, emotional, and functional aspects of QOL, indicating that both were able to identify impairments in other QOL dimensions in addition to the physical domain.

## Key Points

- WHOQOL-BREF and the dermatological-specific Skindex-16 and HRQ-Melasma showed good psychometric performance with satisfactory internal consistency values.
- Only the dermatological-specific instruments tested showed strong correlation between the domains that assess the social, emotional, and functional aspects of quality of life, highlighting their sensitivity to identify impairments in quality of life domains other than the physical domain.
- Specific multidimensional instruments should be used to assess skin disorders related quality of life, when available.
- Skindex-16 and HRQ-Melasma showed superior information on items in most domains.

## Background

Melasma is a pigmentary skin disorder characterized by the presence of hyperchromia, asymptomatic and symmetrical macules in the skin [1, 2]. These macules molecules are formed due to local hypermelanogenesis and typically appear on the face in central locations such as cheeks, chin, nose, and upper lip [3]. In addition, it can occur in other visible body parts such as the neck and upper limbs [2].

Although the exact prevalence of melasma is unknown, several factors have been linked to the development of such macules, including exposure to ultraviolet rays, genetic predisposition, phototoxic drugs, pregnancy, and the use of oral contraceptives [4, 5]. Thyroid issues are also mentioned in the literature as a possible risk factor for melasma [3]. However, because it usually appears after periods of sunlight exposure, it is thought that contact with solar radiation is one of the primary factors in its development [6]. Furthermore, women of reproductive age are the most affected by melasma, indicating that sexual hormones play a decisive role in the pathophysiology of the disease [7, 8].

Although this disease is not physically debilitating, contagious, or life-threatening, evidence shows that melasma has a negative impact on patients' quality of life [9, 10]. This is due to the impairment of appearance when manifesting itself in highly visible places, which can affect self-image, self-esteem, and contribute to the development of negative feelings [11, 12].

According to a study conducted in Latin America, most patients affected by melasma had psychiatric disorders, with depression being the most common [13]. The World Health Organization (WHO) defines quality of life (QOL) as an individual's assessment of his or her own life in relation to his or her goals, expectations, standards, and concerns while considering the cultural context and the values in which it is embedded [14]. Therefore, understanding QOL is critical to comprehend a patient's state of health [3], as it may not be limited to the severity of the disease or the intensity of the lesions [15]. Consequently, using generic or specific instruments that perform the most reliable QOL measurement becomes increasingly important [16].

Generic instruments, such as WHOQOL-BREF, address multidimensional aspects of a patient's life, such as social integration, physical security, mobility, and body image, and can be applied to healthy persons or people affected by a disease [14, 17]. There are also generic instruments for specific health conditions, such as the SKINDEX-16, a generic dermatological tool used in individuals with skin disorders [18]. In contrast, disease-specific instruments focus on a specific area of interest, detecting biological and psychosocial aspects of a specific condition. For the purpose of this study, the HRQ-Melasma was selected to assess the disease-specific QOL as this instrument was developed specifically for individuals with melasma [1]. The advantage of these instruments is that they are sensitive enough to detect specific aspects of a disease's impact on the QOL [19].

For instruments to be recognized as scientifically robust, they must provide accurate, valid, and interpretable data [20]. The quality of the information they provide is largely determined by their psychometric properties, which are measured by their reliability and validity [21, 22]. However, there is a lack in the dermatological literature of studies comparing generic and specific QOL instruments aiming to understand the informative gain in the QOL assessment. Another gap in knowledge relates to the lack of research comparing the performance of a specific against a generic skin disorder QOL instrument to assess the real benefit of using such instruments. As melasma is common in clinical practice, we decided to use the available multidimensional instruments i.e. HRQ-Melasma, Skindex-16, and WOOQOL-BREF.

Therefore, the main aim of this study was to compare the psychometric performance of generic and specific melasma-related quality of life instruments to contribute to the advancement of knowledge in this field. We hypothesized that specific instruments are more sensitive in assessing dermatological related QOL.

## Methods

This was a cross-sectional study with 150 melasma patients attending the dermatology outpatient clinic at the Clinical Hospital of the School of Medicine, São Paulo State University (UNESP), Brazil.

The following eligibility criteria were adopted: patients of both sexes with melasma clinically diagnosed by a qualified dermatologist, aged 18 or older in outpatient care, emotionally capable of responding to the questionnaire, and who agreed to participate in the research. Participants who did not complete the data collection instrument were excluded.

Each participant completed their questionnaire in a private room, individually. The research team clearly stated that refusing to participate in the study would not jeopardize the continuation of their treatment. Data collection occurred between November 2017 and December 2018 using a questionnaire consisting of sociodemographic data, WHOQOL-BREF, Skindex-16, and HRQ-Melasma.

### Generic and specific quality of life instruments

WHOQOL-BREF is a generic instrument that contains 26 items addressing four domains of a patient's life, including physical, psychological, personal relationships, and the environment. The responses are based on a Likert scale of 1-5, with regards to severity in the past two weeks, with the higher score indicating a higher quality of life [23] Table 1.

**Table 1:** Domains and items of the WHOQOL-BREF

Domain	Facets incorporated within domains
Physical health	Activities of daily living Dependence on medicinal substances and medical aids Energy and fatigue Mobility Pain and discomfort Sleep and rest Work Capacity
Psychological	Bodily image and appearance Negative feelings Positive feelings Self-esteem Spirituality/Religion/Personal beliefs Thinking, learning, memory, and concentration
Social relations	Personal relationships Social support Sexual activity
Environment	Financial resources Freedom, physical safety and security Health and social care: accessibility and quality Home environment Opportunities for acquiring new information and skills Participation in and opportunities for recreation/leisure activities Physical environment (pollution/noise/traffic/climate) Transport

Fonte: The WHOQOL Group (1998b) [41]

The Skindex-16 is a multidomain instrument in which answers are given on a 7-point Likert scale, ranging from 0 (never bothered) to 6 (always bothered), based on how often a patient was worried by their skin condition over the previous seven days. It consists of 16 items that encompass three domains: symptoms, emotions, and functionality. All responses are transformed on a linear scale ranging from 0 to 100 points. Scores for each of the three domains are calculated. A higher value indicates a lower quality of life. This instrument has been translated and culturally adapted for use in the Portuguese language [18].

The HRQ-Melasma is a specific instrument to assess the QOL of people suffering from melasma [1]. It has 19 items and reports on the following dimensions: physical/appearance, social/professional, psychological, and treatment [1]. Responses are given on a 5-point Likert scale, ranging from 0 (never applies) to 4 (always), based on how often a patient felt upset or had his routine altered by the males in the last 30 days. Individuals are categorized into the following categories: <15 (not affected); 16-35 (slightly affected); 36-50 (moderately affected); 51-65 (very affected); >65 (extremely affected) [1].

The average time to complete the questionnaire was 20 minutes. All instruments were answered within this time.

### Statistical analysis

Initially, all variables were analyzed descriptively. The score of each of the instrument domains was assessed according to their median, 25^th^ and 75^th^ percentiles (p25 and p75), since they were not normally distributed (Shapiro-Wilk test, p<0.05).

The alpha coefficient of Cronbach's alpha was used to assess internal consistency and values greater than 0.7 [24] were considered significant. The Spearman coefficient was used to analyze the correlation between instrument domains, and it should be greater than 0.7 to indicate a strong correlation [25].

The information in the construct domains was evaluated using the Multi-Array Item Response Theory, and values above 1.2 [26] were considered appropriate. This test was performed using the R software, mirt package. The other analyses were carried out using the IBM SPSS program, version 25. The level of significance adopted was 5%.

The sample size was calculated using the recommendation of a minimum sample size of 150 participants for Item Response Theory-based psychometric studies [27].

The Research Ethics Committee approved the project of the Sao Paulo State University (Protocol n^o^ 2.392.601), and the participants signed a written term of free and informed consent. We confirm that all methods were performed in accordance with the relevant guidelines and regulations.

This manuscript followed the Strengthening the Reporting of Observational Studies in Epidemiology (STROBE) [28].

## Results

The final analytical sample comprised 150 patients with melasma, since there was no refusal. Overall, there were 147 (98%) female participants, with an average age of 41.8 (±8.7) years. Most were married/living with a partner, had a higher level of education, and had a monthly household income between $191 and $570. Most patients reported that they worked indoors, i.e., without sun exposure.

Their occupation types included nursing technician, shop assistant, cleaner, and household service manager. Most participants have been in treatment for between one and five years, and the average age of melasma emergence was 29.9 years (±8.4). In terms of lifestyle, 15% said they were smokers, and 64% drank alcohol. The average body mass index (BMI) was 26.2 (±5.5) kg/m^2^, indicating overweight (Table 2).

**Table 2:** Sociodemographic and clinical characteristics of the participants (*n *=150)

Variable	N (%)
**Sex**	
Female	147 (2.0)
Male	3 (98.0)
**Age (years)**^a^	41.8 (8.7)
**Age of melasma (years)** ^a^	29.9 (8.4)
**Occupation type**	
No sun exposure	145 (97.0)
Sun exposure	5 (3.0)
**Marital status**	
Single	32 (21.0)
Married/Coupled	89 (59.0)
Separate/widowed	29 (10.0)
**Level of education**	
Illiterate	2 (1.0)
Primary school complete	32 (21.0)
High school complete	57 (38.0)
University complete	59 (39.0)
**Monthly household income (USD)**	
Up to $190	21 (14.0)
$191 to $570	64 (43.0)
$571 to $950	22 (15.0)
> $950	43 (29.0)
**Melasma – length of treatment**	
No treatment	8 (5.0)
Less than 1 year ago	41 (27.0)
Between 1 and 5 years	46 (31.0)
Longer than 5 years	55 (37.0)
**Smoking status**	
Yes	15 (10.0)
No	135 (90)
**Alcohol consumption**	
Yes	64 (43.0)
No	86 (57.0)
**Hypertension**	
Yes	23 (15.0)
No	127 (85.0)
**Diabetes**	
Yes	6 (4.0)
No	144 (96.0)
**Dyslipidemia**	
Yes	23 (15.0)
No	127 (85.0)
**BMI**^a^	26.2 (5.5)

^a^ Average (standard deviation) BMI body mass index.

The median (p25-p75) score for each of the WHOQOL-BREF, Skindex-16, and HRQ-Melasma domains are presented in Table 3. The physical domain of WHOQOL-BREF had the highest median (3.9), followed by the emotional domain on Skindex-16 (81) and the psychological domain of HRQ-Melasma [19]. These parameters indicate that quality of life was regular in the physical domain of WHOQOL-BREF, worse in the emotional domain on Skindex-16 and slightly affected in the psychological domain of HRQ-Melasma.

**Table 3:** Distribution of the median score of the WHOQOL-BREF, SKINDEX-16, and HRQ-Melasma domains

Instrument	Median score	p25-p75^a^
**WHOQOL-BREF**		
Physical	3.9	3.3-4.3
Psychological	3.5	3.0-4.0
Personal relations	3.7	3.0-4.0
Environment	3.5	3.1-3.9
**SKINDEX-16**		
Physical	4	0-25
Emotional	81	51-98
Functional	30	0-70
**HRQ-Melasma**		
Physical/Appearance	7	4-10
Social/Professional	3	0-10
Psychological	19	11-27
Treatment	4	2-7

^a^ 25% percentile and 75% percentile.

Table 4 shows the internal consistency values of the WHOQOL-BREF, Skindex-16, HRQ-Melasma domains. It is noted that for all three instruments analyzed, all areas had satisfactory internal consistency (>0.7). In WHOQOL-BREF, the psychological domain had the highest Cronbach alpha value (0.87), and the domains personal relations and environment had the lowest (0.80). For Skindex-16, the domains with the highest internal consistency were the emotional and the functional (0.93), while physical had a lower consistency (0.79). Finally, in HRQ-Melasma, the psychological domain was the most consistent (0.95). However, the treatment had the lower consistency (0.81).

**Table 4:** Internal consistency (Cronbach alpha) for WHOQOL-BREF, SKINDEX-16, HRQ-Melasma domains

Instrument	Cronbach's alpha	Number of items
**WHOQOL-BREF**		
Physical	0.85	7
Psychological	0.87	6
Personal relations	0.80	3
Environment	0.80	8
**SKINDEX-16**		
Physical	0.79	4
Emotional	0.93	7
Functional	0.93	5
**HRQ-Melasma**		
Physical/Appearance	0.86	3
Social/Professional	0.92	5
Psychological	0.95	8
Treatment	0.81	3

Correlations between the domains of WHOQOL-BREF, Skindex-16, and HRQ-melasma are presented in Table 5. The domains that are shared by all instruments were chosen for this analysis. The environment domain of WHOQOL-BREF cannot be correlated with any other field because it addresses aspects of a patient's life that are not assessed by the other instruments. Similarly, the HRQ-Melasma treatment domain is also not represented in the other questionnaires and hence, not included in the analysis.

**Table 5:** Spearman correlation coefficients (*p*-value) among SKINDEX-16, WHOQOL-BREF, HRQ-Melasma and their domains

Variable		W-Phys	W-Psy	W-PRel	W-Envir	SK-Phys	SK-Emot	SK-Funct	H-Phys	H-Soc	H-Psy
WHOO-QOL-BREF	*rho*	0.565									
Psychological	*p*	0.000									
WHOO-QOL-BREF	*rho*	0.377	0.607								
Personal relations	*p*	0.000	0.000								
WHOO-QOL-BREF	*rho*	0.500	0.580	0.443							
Environmental	*p*	0.000	0.000	0.000							
SKINDEX-16	*rho*	-0.318	-0.321	-0.220	-0.352						
Physical	*p*	0.073	0.062	0.007	0.010						
SKINDEX-16	*rho*	-0.117	-0.379	-0.143	-0.308	0.351					
Emotional	*p*	0.152	0.000	0.081	0.000	0.000					
SKINDEX-16	*rho*	-0.234	-0.467	-0.302	-0.471	0.400	0.720				
Functional	*p*	0.004	0.000	0.177	0.000	0.000	0.000				
HRQ-Melasma	*rho*	-0.240	-0.435	-0.169	-0.326	0.313	0.717	0.681			
Physical/Appearance	*p*	0.003	0.000	0.038	0.046	0.096	0.000	0.000			
HRQ-Melasma	*rho*	-0.206	-0.441	-0.267	-0.443	0.391	0.643	0.780	0.705		
Social/Professional	*p*	0.011	0.000	0.000	0.000	0.001	0.000	0.000	0.000		
HRQ-Melasma	*rho*	-0.190	-0.408	-0.199	-0.364	0.335	0.808	0.749	0.788	0.778	
Psychological	*p*	0.020	0.000	0.015	0.005	0.029	0.000	0.000	0.000	0.000	
HRQ-Melasma	*rho*	0.002	-0.066	-0.144	-0.110	0.186	0.435	0.337	0.306	0.321	0.419
Treatment	*p*	0.983	0.425	0.079	0.182	0.023	0.000	0.025	0.139	0.063	0.000

*W-Phys* WHOQOL-BREF - Physical Domain, *W-Psy* WHOQOL-BREF - Psychological Domain, *W-PRel* WHOQOL-BREF - Personal Relations Domain, *W-Envir* WHOQOL-BREF -Enviromemental Domain

*SK-Phys* SKINDEX-16 - Physical Domain, *SK-Emot* SKINDEX-16 - Emotional Domain, *SK-Funct* SKINDEX-16 - Functional Domain

*H-Phys* HRQ Melasma - Physical/Appearance Domain, *H-Soc* HRQ Melasma - Social/Professional Domain, *H-Psy* HRQ Melasma -Psychological Domain

The negative correlations values of WHOQOL-BREF dimensions are due to the reverse direction of the magnitude of the scale. The correlations values between WHOQOL-BREF's domains and Skindex-16 and HRQ-Melasma were the lowest ones. The physical domain correlated poorly with the physical domain of Skindex-16 (-0.31) and did not correlate with the physical domain/appearance of HRQ-Melasma (-0.24). The correlation between the area personal relations with the functional and social/professional obtained coefficients of (-0.30) and (-0.26), respectively, showing a negligible correlation. The psychological domain correlated with the emotional domain of Skindex-16 (-0.37) and the psychological domain of the HRQ-Melasma (-0.40), indicating a weak correlation in both.

When comparing the physical domain of the two dermatological instruments tested, HRQ-Melasma and Skindex-16, there was a weak correlation (0.31). However, there was a strong correlation (0.80) between the emotional domain of Skindex-16 and the psychological domain of the HRQ-Melasma. Similarly, the functional domains of Skindex-16 and social/professional of HRQ-Melasma showed a strong correlation (0.78).

The informative level of the WHOQOL-BREF, Skindex-16, and HRQ-Melasma domains according to the Item Response Theory is shown in Table 6.

**Table 6:** Item response theory for WHOQOL-BREF, SKINDEX-16 and HRQ-Melasma

	Information	Number of items	Corrected information
**WHOQOL-BREF**			
Physical	5.31	7	0.75
Psychological	6.87	6	1.14
Personal relations	3.63	3	1.21
Environment	3.67	8	0.61
**SKINDEX-16**			
Physical	2.52	4	0.63
Emotional	23.69	7	3.38
Functional	20.52	5	4.10
**HRQ-Melasma**			
Physical/Appearance	6.51	3	2.17
Social/Professional	14.54	5	2.90
Psychological	20.09	8	2.51
Treatment	4.06	3	1.35

We found that the physical, psychological, and environmental domains of WHOQOL-BREF, as well as the physical from Skindex-16, did not provide adequate information based on the information corrected by the number of items (values below 1.2). HRQ-Melasma, on the other hand, has shown satisfactory results in all areas.

## Discussion

To the best of our knowledge, this is the first study to compare generic and specific instruments for assessing melasma-related quality of life. Our main findings showed that specific instruments, in addition to the physical domain, have greater sensitivity to identify impairments in other quality of life domains.

Although melasma can affect both sexes, the literature indicates that its prevalence is higher in women of reproductive age due to the activation of melanocytes by female sexual hormones [4, 29], which is consistent with the findings of this study.

In the present study, the comparative analyses of the three QOL instruments was based on internal consistency, validity and item information using the Item Response Theory. The item information was the most relevant aspect for the present analysis since the available evidence shows that responsiveness, a measure of longitudinal validity, is not always considered by all researchers to be a psychometric property. However, current definitions highlight the importance of such measure to assess the validity of changes in score of an instrument [30–32].

On the other hand, in the Item Response Theory, standard responses of an individual to a particular group of items provide the base to estimate the latent traits, by allowing a better use of the information gathered. This approach allows not only the classification of individuals in relation to their latent traits but also to collect information on an instrument as a whole and, particularly, item by item [33]. The Item Response Theory should not be seen as a method to replace the classic theory but as a complementary analytical tool [34].

In terms of Skindex-16's consistency, our study found consistent values for all domains, with higher emotional scores (0.93). Cronbach alpha was 0.86, 0.93, and 0.88 for the Physical, Emotional, and Functional domains, respectively, in a study using Skindex-16 in patients with various dermatoses [18].

The HRQ-Melasma instrument demonstrated high internal consistency in the development study, with values of 0.88, 0.91, 0.93, and 0.73 for the areas of Physical/Appearance, Social/Professional, Psychological, and Treatment, respectively [1]. Similarly, the present study observed Cronbach's alpha above 0.7 for these domains and the psychological domain with a higher coefficient (0.95). This study found that the domain with the highest Cronbach alpha value was related to psychological aspects, in addition to reaffirming significant internal consistency in the three instruments evaluated. This supports the notion that melasma is a disease that significantly impacts self-image, as evidenced by the literature [1, 3].

The WHOQOL-BREF' Personal Relations' and 'Environment' domains, on the other hand, had the lowest Cronbach alpha values. This could be attributed to the low sensitivity of this instrument in detecting the social and environmental impacts on melasma. The physical domain of Skindex-16 also supports the notion that melasma is a disease with a low physical impact that poses no threat to the patient's life [1]. However, when comparing the internal consistency of the physical domains of the three instruments analyzed, we found that HRQ-Melasma shows the highest Cronbach's alpha, demonstrating that a specific instrument has a greater correlation between the items in the domains, making its internal consistency higher and with this, increasing its capacity to perceive the inconvenience caused by the melasma.

Regarding instrument correlation, there is a scarcity of comparative studies between generic and specific instruments in the dermatological research area. The lack of correlation between WHOQOL-BREF, Skindex-16, and HRQ-Melasma highlights the fact that WHOQOL-BREF did not capture the impact of melasma on quality of life as well as the other instruments. This can be attributed to the fact that WHOQOL-BREF is a generic instrument for several areas, with no questions directed toward melasma. Therefore, systemic conditions may outweigh the conditions affected by dermatosis.

A strong correlation was found between the emotional and social domains of Skindex-16 and HRQ-Melasma. This indicates that both instruments managed to capture the inconvenience caused by melasma in these aspects. However, there is no correlation between the physical domains of the instruments, indicating that melasma has physical implications that are not consistent and vary greatly between patients [35].

Concerning the information on the items that comprise the three instruments, we found that the generic instrument, i.e., WHOQOL-BREF revealed domains that were less informative than the others, indicating its reduced capacity for capturing the true impact of melasma on one's quality of life. However, in comparing common domains between the three instruments tested, such as the Psychological WHOQOL-BREF, Emotional of Skindex-16, and Psychological of the HRQ-Melasma, the greatest level of information came from Skindex-16. Similarly, for the Personal Relations area of WHOQOL-BREF, Functional of Skindex-16, and Social/Professional of HRQ-Melasma, the greatest information was provided by Skindex-16, highlighting the sensitivity of this instrument to detect changes in the quality of life caused by melasma.

Melasma is a chronic dermatological disease that impacts significantly on psychological and emotional aspects affecting one’s quality of life. Furthermore, dermatological diseases that are visible are associated to higher psychiatric morbidities [36, 37]. Patients with facial Melasma frequently report feelings of frustration, shame, low self-esteem, anxiety and depression [38–40].

### Limitations

Finally, it is important to highlight that the lack of similar comparative studies on quality of life instruments made difficult for us to compare and discuss our key findings. However, it also showed that further studies are needed in this area. Another potential limitation of the present study relates to the fact that the questionnaire was applied only once and, therefore, not allowing us to perform both responsiveness and temporal stability analyses.

## Conclusions

Overall, the three quality of life instruments tested showed good psychometric performance with satisfactory internal consistency values. However, only the dermatological specific instruments showed a strong correlation between domains that assess the social, emotional, and functional aspects of QOL, highlighting the sensitivity of both to identify impairments in other domains of quality of life, besides the physical one. Furthermore, Skindex-16 and HRQ-Melasma showed superior information on items in most domains.

## Data Availability

The data that support the findings of this study are stored in a research server at the São Paulo State University (UNESP) medical school, Botucatu, Brazil. An anonymous dataset in Excel format can be made available. For data access requests, please contact the Chair of the Ethics Committee of the Faculty of Medicine of Botucatu, Dr Silvana Andreia Molina Lima, e-mail: cep.fmb@unesp.br, Botucatu, Sao Paulo, ZIP Code: 18618-000, Brazil, Telephone no. +55 14 38801608.
